# What Does Personality Mean in the Context of Mental Health? A Topic Modeling Approach Based on Abstracts Published in Pubmed Over the Last 5 Years

**DOI:** 10.3389/fpsyt.2019.00938

**Published:** 2020-01-09

**Authors:** Raffaele Sperandeo, Giovanni Messina, Daniela Iennaco, Francesco Sessa, Vincenzo Russo, Rita Polito, Vincenzo Monda, Marcellino Monda, Antonietta Messina, Lucia Luciana Mosca, Laura Mosca, Silvia Dell'Orco, Enrico Moretto, Elena Gigante, Antonello Chiacchio, Chiara Scognamiglio, Marco Carotenuto, Nelson Mauro Maldonato

**Affiliations:** ^1^ SiPGI–Postgraduate School of Integrated Gestalt Psychotherapy, Torre Annunziata, Italy; ^2^ Department of Clinical and Experimental Medicine, University of Foggia, Foggia, Italy; ^3^ Department of Ophthalmology, University of Foggia, Foggia, Italy; ^4^ Department of Environmental, Biological and Pharmaceutical Sciences and Technologies, University of Campania Luigi Vanvitelli, Caserta, Italy; ^5^ Department of Experimental Medicine, Section of Human Physiology and Unit of Dietetic and Sport Medicine, University of Campania Luigi Vanvitelli, Naples, Italy; ^6^ Department of Mental Health, Physical and Preventive Medicine, Clinic of Child and Adolescent Neuropsychiatry, University of Campania Luigi Vanvitelli, Naples, Italy; ^7^ Department of Neurosciences and Reproductive and Odontostomatological Sciences, University of Naples Federico II, Naples, Italy

**Keywords:** personality disorder, mental health, personality trait, topic analysis, mental health promotion

## Abstract

Personality disorders (PDs) are one of the major problems for the organization of public health systems. Deepening the link between personality traits and psychopathological drifts, it seems increasingly essential for the often dramatic repercussions that PDs have on social contexts. Some of these disorders, such as borderline PD, antisocial PD, in their most tragic expression, are the basis of problems related to crime, sexual violence, abuse, and mistreatment of minors. Many authors propose a dimensional classification of personality pathology, which has received empirical support from numerous studies over the last 20 years based on more robust theoretical principles than those applied to current nosography. The present study investigates the nature of the research carried out in the last years on the personality in the clinical field exploring the contents of current research on personality relapses, evaluating, on the one hand, the emerging areas of greatest interest and others, those that they stopped generating sufficient motivations in scholars. This study evaluates text patterns regarding how the terms “personality” and “mental health” are used in titles and abstracts published in PubMed in the last 5 years. We use a topic analysis: Latent Dirichlet Allocation that expresses every report as a probabilistic distribution of latent topics that are represented as a probabilistic distribution of words. A total of 7,572 abstracts (from 2012 to 2017) were retrieved from PubMed for the query on “mental health” and “personality.” The study found 30 topics organized in eight hierarchical clusters that describe the type of current research carried out on personality and its clinical relapse. The hierarchical clusters latent themes were the following: social dimensions, clinical aspects, biological issues, clinical history of PD, internalization and externalization symptoms, impulsive behaviors, comorbidities, criminal behaviors. The results indicate that the concept of personality is associated with a wide range of conditions. The study of personality and mental health still proceeds, mainly, according to a practical-clinical approach; too little moves, however, according to an innovative research approach, but the work shows the common commitment of scholars to a new way of dealing with the study of personality.

## Introduction

Interactions between people are governed by patterns of bio-psycho-social processes whose purpose is the adaptation to the environment and which together describe what we define as personality. All people are characterized by their own way of relating to others and adapting to different contexts. This peculiarity can be studied by describing analytically every single function involved. As a set, these functions are routinely referred to as “personality traits.” The models that describe the phenomenology and the evolution are many and often antithetical to each other. This has hitherto prevented the emergence of exhaustive theories capable of systematizing the amount of empirical data resulting from clinical observation and research (1). According to the *DSM-5* a personality disorder (PD) is a constant pattern of inner experience and behavior that deviates markedly from the expectations of the culture to which the individual belongs. These behavioral patterns are substantiated in interpersonal conflicts, difficulties in creating and maintaining intimate relationships, and establishing and achieving reasonable existential goals (2, 3).

In the current state of knowledge, the relationship between personality constructs and PD is a crucial point for understanding the topic. In the international scientific community, a general agreement has been reached in terms of the definition. WHO defines personality as a structured mode of thought, feeling, and behavior—expression of constitutional factors, development, and social experience (*ICD-10*)—which characterizes the type of adaptation and lifestyle of a subject (4). Personality traits describe adaptive behaviors that in their most extreme expressions configure the substrate on which a PD is grafted. For example, low levels of a personality trait defined as “amiability” represent the condition for the development of hostile behavior in social relationships, which is a real symptom of a PD. This does not mean that the presence of idiosyncratic and dysfunctional personality traits is always the cause of clinical problems. Additionally, these traits are present in every person and it is not easy to recognize the point in which they evolve in a PD, generating suffering in the subject and in the people with whom they come into contact. In fact, the transition of personality traits from healthy to dysfunctional and, finally, to pathological is partly determined by context variables and by the demands that the subjects receive in the environments in which they move. These variables are difficult to identify and to correlate with the individual's answers (5–7). Although clinicians and most scholars currently believe that PD symptoms are the product of extreme expression of healthy personality traits (both in the sense of deficiency and excessive manifestation), there is still no satisfactory theoretical model that can support this point of view. The classification of PDs is still based, with small variations, on the categorical model proposed in 1980 by the *DSM-IIIR*.

The categorical classification currently officially included in the *DSM-5* present 10 PDs divided into three clusters. It presents three (among others) repeatedly emphasized limits in the scientific literature: (a) the considerable internal heterogeneity of each disorder, (b) the arbitrariness of the diagnostic threshold chosen for each disorder, (c) the overlap between the different diagnostic categories. These limits make the categorical model of PDs scarcely credible and lead us to believe that they represent artificial constructs with a limited scientific support (8).

This has led many authors to propose a dimensional classification of personality pathology, which has received empirical support from numerous studies over the last 20 years based on more robust theoretical principles than those applied to current nosography. During the development of the fifth edition of the DSM, an attempt was made to find a new classification of mixed (dimensional and categorical) PDs based on the five-factor personality model. This attempt was accepted only by a minority of the authors and is today relegated to the appendix of the manual. However, it clearly expresses the emerging position that could solve many current nosographic and conceptual problems concerning PDs: the pathology of personality consists on the presence of extreme manifestations of the traits of the normal personality (9). In the categorical formulation (i.e. with arbitrary threshold criteria), PDs are diagnosed in 30% of those affected by a psychiatric disorder of which they significantly influence both the course and the prognosis. In the light of current literature, dysfunctional personality traits that do not exceed the categorical diagnostic threshold are likely to be present in all subjects with a psychiatric disorder and may be considered as risk or protection factors for the onset and course of mental illness and suicide (10). The presence of dysfunctional personality traits can both act as a trigger for the development of anxiety, depressive, or psychotic symptoms and make it difficult to manage the symptoms of psychiatric disorders. Moreover, because PDs are strongly sensitive to environmental and relational contexts, it could be easier to insert the dimensions of the personality within relational dynamics and to recognize the environmental variables at the origin of the pathological processes (11).

If this new concept of PD was able to overcome the tests of reliability and validity, it would have enormous clinical repercussions and could represent a real revolution for treatment protocols and prevention. In fact currently the therapeutic approach to PDs fails to take into adequate consideration the environmental and relational dimensions in which the individual is immersed and which have a heavy effect on the development and progression of character pathologies. This lack reduces the efficiency of therapeutic programs. The more flexible and focused nature of the dimensional concepts of the personality, in their extreme expressions, can be effectively related, more than categorical disorders, to the specific environmental conditions and relational dynamics of the patients.

The present study investigates the nature of research conducted in recent years regarding personality in the clinical field and is inscribed in a crucial season of the flowering of new approaches to the study of personality, which appear increasingly indispensable for the often dramatic repercussions that the PD have on public mental health and social welfare. In their most tragic expression, such disorders are, in fact, the basis of problems related to crime, sexual violence, abuse, and mistreatment of minors. It is now well established that a PD frequently causes serious relational and family problems, dependence on alcohol or drugs, situations of social withdrawal, loneliness, and depression (12).

In recent years, scientific production on the clinical relapses of personality traits have produced datasets of extreme interest and has expanded the semantic field of personality-related concepts. Both on the theoretical and the empirical elaboration side, it is increasingly necessary to recognize the new emerging connections (13).

A useful approach to achieve this goal is to evaluate the frequency of scientific terms used and the way in which the same terms are aggregated in research on personality and its disorders. The statistical technique used in the present study Latent Dirichlet Allocation allows to grasp and explain, through a hierarchical system of aggregation of words, the latent conceptual connections between the terms that occur with greater frequency in the most recent scientific productions (14).

In the study carried out, the scientific terms present in the abstracts published on Medline in the last 5 years were analyzed, with the aim of exploring the contents of current research on the personality relapses, assessing on the one hand the emerging areas of greatest interest and other, those that have stopped generating sufficient motivations in scholars. The study also intends to highlight the presence of new concepts or new connections between the concepts that can bring greater descriptive abilities, thus overcoming the classical definitions of the relationships between personality and mental disorders, finally entering the decisive field of action for the themes in the object.

## Methods

### Study Design

This study evaluates text patterns regarding how the terms “personality” and “mental health” are used in titles and abstracts published in PubMed. These patterns were also investigated in relation to their trends over time analysis, as well as in a hierarchical cluster analysis to examine how they were related to each other. This study is described in accordance with the STROBE (STrengthening the Reporting of OBservational studies in Epidemiology) guidelines STROBE statement includes a checklist of 32 items recommended for inclusion in the reporting of observational studies. These items are related to aspects of study design, sample selection, data collection, analysis, and potential bias (15).

### Dataset

We explored and recovered title and abstracts from publications regarding personality and mental health using the PubMed bibliographic database (https://www.ncbi.nlm.nih.gov/pubmed/). PubMed research (PubMed is a medical database) was carried out with the aim of limiting the sample to studies related to the use of the concept of PDs in the medical field.

Our search involved the following approach: (mental disorders[MH] OR mental health[MH]) AND personality[Title/Abstract] AND (“2012/12/31”[Date - Entrez]: “2017/12/31”[Date - Entrez]) AND has abstract [text]. The textual content was then pre-processed *via* the elimination of frequently- used words (for example, background, aim, method, result, conclusion, information, frequent, context, among, suggest, although, possible, include, article, however, also, shown, later, main, view, within, and find), numerical digits, stop- words, and punctuations. Pre-processing was conducted through the tm (textual content mining) package used in the statistical language R (16).

### Topic Modeling

We made use of a topic model, aimed at determining the main principal subjects from the group of texts for this analysis. Put simply, probabilistic topic models summarize an abstract. A key aspect of developing a topic model is determining the degree of similarity among concepts, defined as a connection between different words that goes beyond its usage and meaning (17). Previous publications have investigated its use in a variety of scenarios, for example in a bibliographic analysis to explore FDA priorities or to evaluate the association between concept in medical notes and genetic information (18). A topic is operationally defined as the likelihood of a group of words being aggregated over a set of phrases. Specifically, we used the Latent Dirichlet Allocation (LDA), a hierarchical Bayesian method where every report may be expressed as a probabilistic distribution of latent topics, and also where a latent topic is represented as a probabilistic distribution of words (14, 19). Topic-file distributions, word-topic distributions, and hidden parameters are then estimated by the model through the observed words and documents. The LDA (Latent Dirichlet Allocation) model is a generative model, used in the study of natural language, which allows to extract topics from a set of source documents and to provide a logical explanation on the similarity of single parts of the documents. The generative process of Latent Dirichlet Allocation is based on the analysis of data contained in the text (text mining). Word combinations are considered to be random variables.

As a first step, we defined the number of topics through a cross-validation method. Briefly, our dataset was split into five randomly selected subsets, where the first subset is used for model training, while the remaining four are used for validation, i.e. using a holdout mechanism. Different numbers of topics extracted from the dataset are extracted and then tested in relation to the perplexity statistical parameter comparing the training value against the holdout samples (20–23). In information theory, perplexity is a measurement of how well a probability distribution or probability model predicts a sample. It may be used to compare probability models. A low perplexity indicates the probability distribution is good at predicting the sample (24).

### Topic Visualization

We recorded the most frequent words in each topic as a representation of subject matter underlying that topic. Word clouds were then included to represent the central concepts for each topic, with font sizes being proportional to the frequency of a word being inside a topic.

### Dynamics and Hierarchical Clustering of Topics

We conducted a trend analysis of each topic's proportion from 2012 to 2017, also implementing a hierarchical clustering analysis based on a topic-word matrix to explore the association among topics.

Hierarchical clustering is a clustering approach that aims to build a hierarchy of clusters. it can be used to examine data distributions and to observe the characteristics of each distribution The matrix content was transformed into a binary format denoting the presence or absence of a word in a given topic. The average linkage method was applied to generate the cluster dendrogram (25), an Euclidean distance being the basis for assessing the range of topics.

## Results

A total of 7,572 abstracts published from 2012 to 2017 were retrieved from PubMed for the query on “mental health” and “personality.”

**Figure 1:**
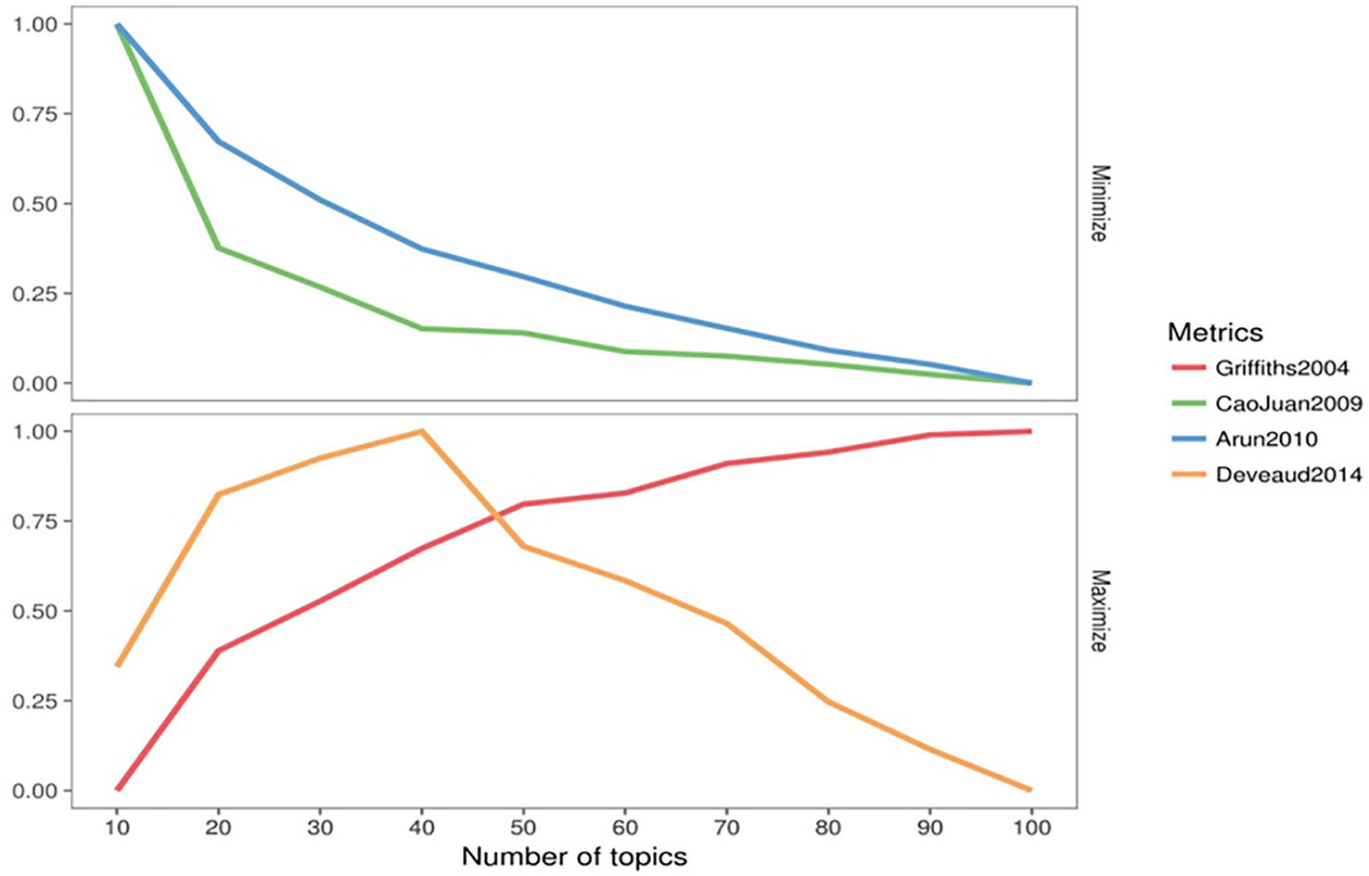
Fit calculation plot of 4 measures/metrics for a range of topics.

**Figure 1** displays the range of the number of topics that can identify the theme comprising a document. The different metrics used in topic estimation agree that a minimum of 20 topics is optimal to discover relevant information from the mental health and personality dataset.

5-fold cross-validation of topic models was performed to choose the optimal number of topics that can describe latent themes in the corpus. The cross-validation was carried out for multiple values (10 to 100) of *k* = number of topics. These results are largely consistent with measures of topic- estimation in **Figure 1**, with a distinct flattening of the cross- validated perplexity metric somewhere between 30 and 60 topics. A lower number of topics results in lumping of distinct themes into a single topic, whereas, increasing the number of topics beyond the 60- topic threshold would result in a recurrence of similar themes distributed across multiple topics. Therefore, we selected an LDA with 30 topics to discover relevant issues in our document corpus.

**Figure 2:**
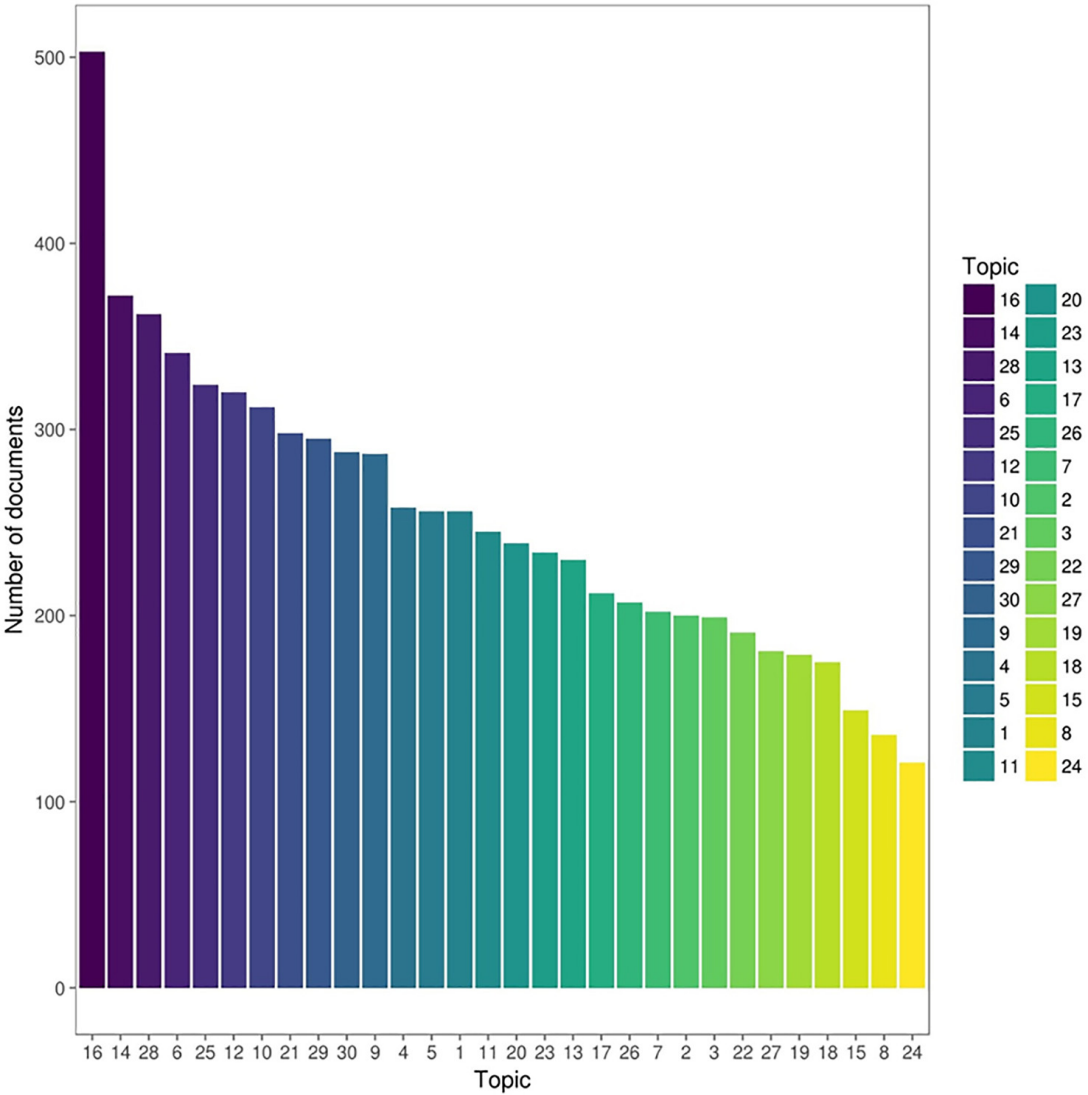
Distributions of assigned top topics with the highest probability for documents abstracts. **1** schizophrenia, obsessions and personality - **2** anxious-depressive disease and personality - **3** qualitative assessment of personality pathology - **4** personality and quality of life - **5** eating behavior disorders and personality - **6** personality and violent behavior - **7** personality impacts on the evolution of psychiatric pathology - **8** gender differences in personality – **9** socialand health needs of psychiatricpatients - **10** impact of genetic factors on temperament and character - **11** suicidal behaviors and personality pathologies - **12** personality and chronic or degenerative pathologies - **13** personality functioning and post-traumatic disorder, dissociative states - **14** personality functioning and substance use disorders - **15** relevance of measurement systems - **16** effectiveness of clinical treatments and psychotherapy - **17** cognitive functions and personality pathologies - **18** therapeutic change and psychotherapy - **19** research methods - **20** categorical classification of psychiatric disorders -**21** biological mechanisms of personality pathologies - **22** relational dimension of the personality - **23** psychological invariants common to the population - **24** scientific evidence in personality theories - **25** categorical psychiatric evaluation model - **26** personality-related concepts and psychic distress - **27** borderline personality disorder - **28** quantitative evaluation tools of personality - **29** personality structure and individual development - **30** emotional affective dimension in personality structure.

The topics extracted from LDA with *k* = 30 topics were named T1-T30. Distribution of assigned topics for the documents is demonstrated in **Figure 2**.

**Figure 3:**
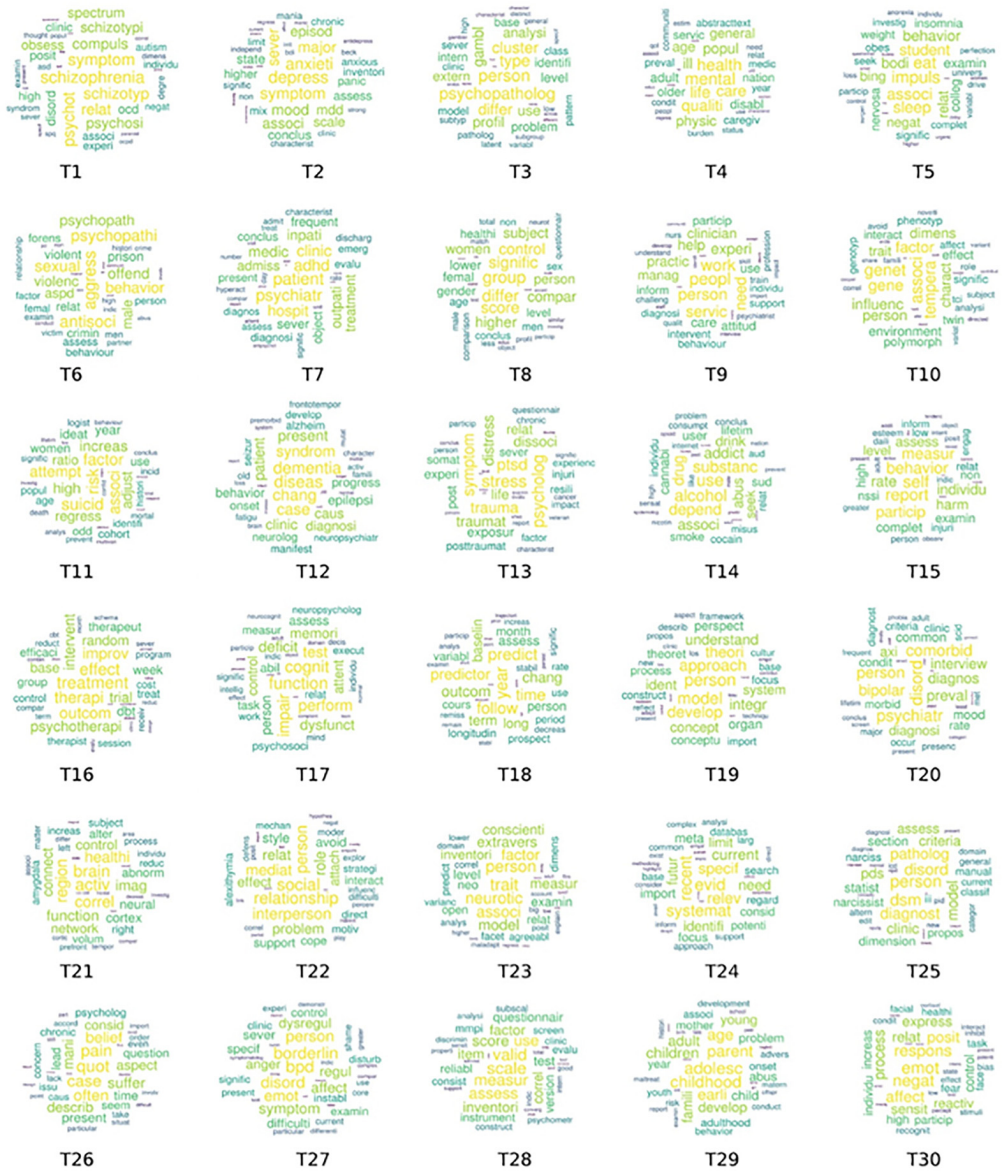
Word clouds for all topics, where asterisk represents any combination. **1** schizophrenia, obsessions and personality - **2** anxious-depressive disease and personality - **3** qualitative assessment of personality pathology - **4** personality and quality of life - **5** eating behavior disorders and personality - **6** personality and violent behavior - **7** personality impacts on the evolution of psychiatric pathology - **8** gender differences in personality - **9** social and health needs of psychiatric patients - **10** impact of genetic factors on temperament and character - **11** suicidal behaviors and personality pathologies - **12** personality and chronic or degenerative pathologies - **13** personality functioning and post-traumatic disorder, dissociative states - **14** personality functioning and substance use disorders - **15** relevance of measurement systems - **16** effectiveness of clinical treatments and psychotherapy - **17** cognitive functions and personality pathologies - **18** therapeutic change and psychotherapy - **19** research methods - **20** categorical classification of psychiatric disorders -**21** biological mechanisms of personality pathologies - **22** relational dimension of the personality - **23** psychological invariants common to the population - **24** scientific evidence in personality theories - **25** categorical psychiatric evaluation model - **26** personality-related concepts and psychic distress - **27** borderline personality disorder - **28** quantitative evaluation tools of personality - **29** personality structure and individual development - **30** emotional affective dimension in personality structure.

The 10 most probable words for each topic are listed in **Table 1** and the corresponding word cloud for each topic is shown in **Figure 3**. A larger size and a more intense color of a particular word implies that it was used more frequently in the corpus of the document being analyzed. For example, in topic T1, the word “schizophrenia” presented the largest frequency and can thus be prominently visualized in the word cloud for T1. These words are represented by their roots. For example, depress can represent instances of words such as depression, depressed, or depressing. Words with high probabilities in each LDA-derived topic were selected to express the dataset.

**Table 1:** The most probable words in the topics of lda with k topics.

	Term 1	Term 2	Term 3	Term 4	Term 5	Term 6	Term 7	Term 8	Term 9	Term 10
**Topic 1**	schizophrenia	symptom	psychot	schizotyp	relat	compuls	Psychosi	schizotypi	spectrum	obsess
**Topic 2**	depress	anxieti	symptom	sever	major	mood	Associ	episod	mdd	scale
**Topic 3**	person	psychopatholog	type	Cluster	differ	gambl	Profil	use	analysi	extern
**Topic 4**	mental	health	life	qualiti	care	ill	Popul	physic	age	general
**Topic 5**	impuls	eat	associ	sleep	student	behavior	Bodi	relat	bing	negat
**Topic 6**	aggress	antisoci	psychopathi	sexual	behavior	offend	Psychopath	violenc	male	aspd
**Topic 7**	patient	psychiatr	adhd	clinic	hospit	medic	inpati	outpati	treatment	admiss
**Topic 8**	group	differ	signific	score	control	higher	compar	subject	women	person
**Topic 9**	peopl	work	person	servic	need	experi	Help	practic	clinician	manag
**Topic 10**	associ	genet	tempera	gene	factor	influenc	person	dimens	charact	traitf
**Topic 11**	risk	suicid	factor	associ	attempt	increas	High	adjust	regress	ratio
**Topic 12**	diseas	dementia	chang	case	syndrom	present	patient	clinic	caus	diagnosi
**Topic 13**	stress	psycholog	ptsd	Symptom	trauma	distress	traumat	clinic	caus	diagnosi
**Topic 14**	use	alcohol	substanc	Depend	drug	addict	associ	abus	drink	seek
**Topic 15**	self	report	behavior	Measur	particip	rate	Individu	harm	assess	level
**Topic 16**	treatment	effect	therapi	Outcom	improv	intervent	psychotherapi	trial	random	base
**Topic 17**	function	cognit	impair	Perform	test	deficit	Dysfunct	memori	attent	control
**Topic 18**	year	predict	follow	time	predictor	outcom	chang	term	baselin	long
**Topic 19**	person	model	approach	Develop	theori	integr	Understand	ident	concept	system
**Topic 20**	disord	psychiatr	comorbid	Person	bipolar	preval	diagnos	interview	axi	diagnosi
**Topic 21**	activ	brain	region	correl	healthi	function	imag	connect	control	network
**Topic 22**	social	relationship	person	interperson	mediat	role	relat	problem	effect	attach
**Topic 23**	trait	person	neurotic	associ	factor	model	Extravers	measur	Conscien	inventori
**Topic 24**	evid	recent	systemat	relev	specif	current	need	futur	identifi	limit
**Topic 25**	person	dsm	disord	Diagnost	patholog	model	clinic	pds	criteria	assess
**Topic 26**	quot	pain	case	often	belief	suffer	mani	describ	aspect	consid
**Topic 27**	bpd	borderlin	disord	erson	emot	symptom	affect	regul	dysregul	anger
**Topic 28**	scale	valid	measur	assess	use	score	Invention	factor	item	correl
**Topic 29**	adolesc	childhood	parent	age	earli	children	adult	famili	develop	young
**Topic 30**	emot	negat	respons	affect	posit	process	relat	express	sensit	reactiv

The results indicate that the concept of personality is associated with a wide range of conditions. For example, Topic 1 associated personality with schizophrenia-related conditions, involving issues related to the personality of these patients, how psychotherapy might be related to personality, schizotypical personality and its relation to personality, and the relationship between personality and schizophrenia.

**Figure 2** demonstrates the five most popular topics among the corpus dataset where T16 pertains to “interventional therapy,” T14 to “substance abuse,” T28 to “research instrument,” T6 to “antisocial personality disorder,” and T25 to “psychiatric assessment.” Along with themes above, the LDA algorithm with 30 topics also uncovered issues such as eating disorder, suicidal behavior, and emotional behavior (26, 27). Finally, our results indicate that many studies focus on assessment, symptoms, and treatment of mental health along with factors including physical, mental, social, genetic, and behavioral aspects, all of which can be influenced by an individual's personality.

Word clouds are a visual representation displaying the content of topics, also demonstrating term frequency count of words in each topic. **Figure 3** demonstrates word clouds for all 30 topics. A larger size of a particular word implies that it was used more frequently in the corpus of the document being analyzed. For example, in topic T1, the word “schizophrenia” presented the largest frequency and can thus be prominently visualized in the word cloud for T1. Similarly, the latent theme for topic 27 is “personality disorder” which can be inferred based on its word cloud with word the “bpd (borderline personality disorder)” having the largest frequency.

**Figure 4:**
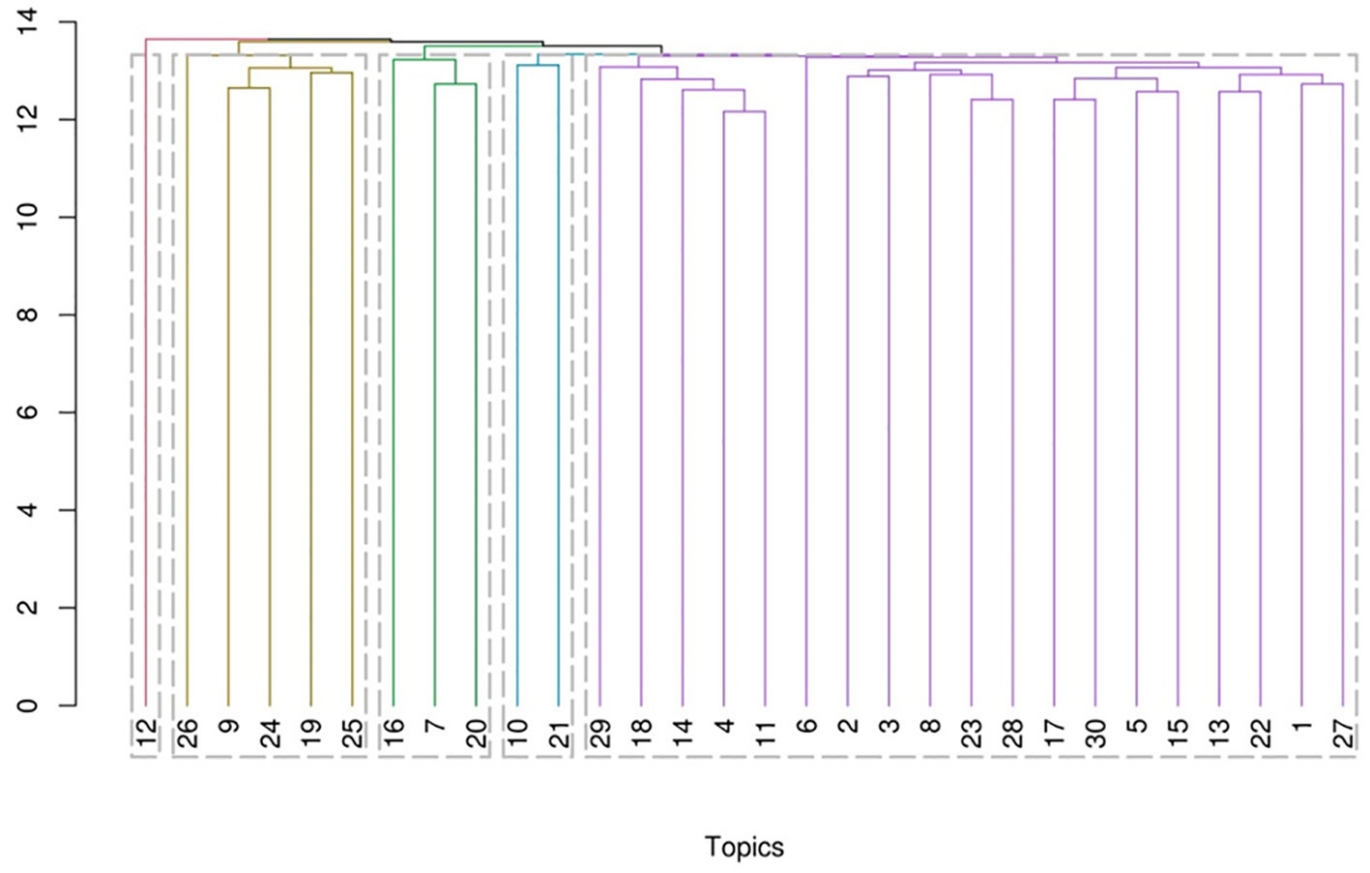
The cluster dendrogram of topics based on hierarchical cluster analysis. **1** schizophrenia, obsessions and personality - **2** anxious-depressive disease and personality - **3** qualitative assessment of personality pathology - **4** personality and quality of life - **5** eating behavior disorders and personality - **6** personality and violent behavior - **7** personality impacts on the evolution of psychiatric pathology - **8** gender differences in personality – **9** socialand health needs of psychiatricpatients - **10** impact of genetic factors on temperament and character - **11** suicidal behaviors and personality pathologies - **12** personality and chronic or degenerative pathologies - **13** personality functioning and post-traumatic disorder, dissociative states - **14** personality functioning and substance use disorders - **15** relevance of measurement systems - **16** effectiveness of clinical treatments and psychotherapy - **17** cognitive functions and personality pathologies - **18** therapeutic change and psychotherapy - **19** research methods - **20** categorical classification of psychiatric disorders -**21** biological mechanisms of personality pathologies - **22** relational dimension of the personality - **23** psychological invariants common to the population - **24** scientific evidence in personality theories - **25** categorical psychiatric evaluation model - **26** personality-related concepts and psychic distress - **27** borderline personality disorder - **28** quantitative evaluation tools of personality - **29** personality structure and individual development - **30** emotional affective dimension in personality structure.

The cluster dendrogram of all 30 topics is demonstrated in **Figure 4**. The topics referring to related themes are clustered together since they are represented by similar words. The similarity of two topics is indicated by the point on the vertical axis where their lines are joined; the lower that point is, the more similar the topics are. For example, two of the topics suggested by the dendrogram to be relatively similar are T4 and T11, T4 focusing on “mental health care” and T11 relates to “suicidal behavior.” Even though these topics do not share any common words among their top 10 most probable words (**Table 1**) the connection between them is immediately evident since suicidal behavior and mental health are intimately connected. Similarly, T23 and T28 are closely connected as they pertain to personality assessment” and “research instrument,” respectively.

### Analysis of the Emerged Clusters

In the dendrogram based on the hierarchical analysis of the clusters, eight topic groups are identified. Analyzing it from a psychopathological and clinical point of view, we have identified a latent theme for each of these groups. Below we will discuss the meaning of the latent themes of the eight clusters and the internal articulation of the topics that belong to them.

The first group of topics brings together the social dimensions of personality pathology and the suffering associated with this type of pathology with theoretical models, methods of scientific investigation and psychiatric diagnostic methods. The latent tract to these topics appears to be the “conceptual revision of the personality in its pathological expressions” with the need to review the current nosography and base it on a solid empirical basis, drawing on the theoretical models of personality (28). In detail:

- Topic 9 focuses on the social and health needs of psychiatric patients and it is present in almost 300 different research works.
- In topic 19 the central concept concerns research methods; this topic is present in little more than 180 studies.
- Topic 24 is present in little more than 120 studies and expresses the need for solid scientific evidence in personality theories.
- Topic 25 is present in more than 300 studies and expresses interest in redefining and overcoming the categorical psychiatric evaluation model.
- Topic 26 is present in approximately 220 studies and clarify how personality- related concepts are present in many studies of psychic distress.

The second group deals with research on mainly clinical aspects such as hospitalization, history of illness, outpatient treatment, health care, type of therapeutic interventions, efficacy of treatments, co-morbidities, and diagnostic methods. The latent trait underlying these topics is the “clinical practice” that has a profound need to clarify effective methods of intervention, to provide itself with consistently organized health structures and effective and valid diagnostic methods (29). In detail:

- Topic 7 clarify the existence of an area of research that points out the need to investigate how the personality impacts on the evolution of psychiatric pathology in general and on health care. This topic is present in about 200 studies.
- Topic 16 is present in more than 500 studies and expresses the interest of the scientific community on the effectiveness of clinical treatments and in particular on the effectiveness of psychotherapy.
- Topic 20 is present in approximately 250 studies and expresses interest in the study of categorical classification of psychiatric disorders.

The third group is composed of about 600 studies containing biological investigation topics on the personality in both the genetic and neurobiological sense. “The biological cause of personality disorders” is the latent concept of these two topics. This is a field of preferential investigation for diseases such as antisocial disorder (due to heredity), borderline disorder (due to its relation with impulse control), and schizoid, schizotypal, and paranoid disorders (due to the relation with psychosis that scholars implicitly attribute to these disorders) (30). In detail:

- The topic 10 describes the researchers' interest regarding the impact of genetic factors on temperament and character, in dimensional terms, and in personality traits. The relevance of this topic is shown by fact that it is present in more than 300 studies.
- Topic 21 is present in less than 300 studies and expresses interest in the biological mechanisms of personality pathologies (26).

In the fourth group of topics the study of the evolution of personality development, the analysis of the clinical history of PD and the response to psychotherapeutic treatments are linked to investigations on the management systems of mental health of the territory, on suicidal risk and on the use of drugs. The latent concept that holds these topics together is the knowledge of the process of development and of the “natural history of the pathology of personality.” For this purpose it is necessary to activate follow-up studies to evaluate the autobiographical antecedents and proximal causes of PD, to know clearly the relationship between PD and substance abuse disorders, the risk of suicide, and the quality of life of the subjects (31). In detail:

- Topic 4 refers to the importance of personality as a key element for the quality of life of patients. It is present of this in over 250 studies in the last 5 years.
- Topic 11 focuses on the importance of research on suicidal behaviors as consequences of personality pathologies. This topic is present in approximately 300 studies.
- The topic 14 expresses the high degree of co-morbidity between personality functioning and substance use disorders. This theme is found in over 350 works.
- Topic 18 is present in approximately 180 studies and expresses interest in longitudinal follow-up studies useful for evaluating change following psychotherapy.
- Topic 29 is present in about 300 studies and expresses the interest for the definition of the emergence of the personality structure, in the course of individual development.

The fifth group of topics relates the pathological personality and individual and gender differences with the manifestation of pathologies from internalization and externalization. This personality approach is a factorial type and uses quantitative and semi-quantitative evaluation tools. The latent concept underlying this group of topics is the attempt to distinguish typical symptoms from “manifestations associated with personality pathologies.” In this way, the interrelationship between psychometric studies that produce tools in order to assess the factorial structure of the personality and studies that investigate the relationship between pathological personality and disorders that frequently associate with it without constituting the skeleton, is clarified (32). In detail:

- Topic 2 is present in about 200 studies and highlights the interest of research for the connection between anxious-depressive disease and personality.
- Topic 3 expresses the interest both for the qualitative assessment of personality pathology and for categorical nosography. This topic is present in more than 250 works.
- Topic 8 express the interest in the effects of gender differences on personality. this topic is found in just over 100 studies.
- Topic 23 is present in approximately 220 studies and expresses interest for psychological invariants common to a large portion of the population.
- In the topic 28 is present in about 350 studies and expresses the interest for the study of personality through the use of quantitative evaluation tools.

The sixth group of topics describes personality in relation to impulsive issues, eating disorders, neuropsychological functions, and affective aspects. Also in this case the investigations are accompanied by the critical analysis of the psychological reagents. The latent concept to this group is the “neuropsychological dimension of the pathology of personality” that seems to explain the impulsive pathology and the control of emotions and of the alimentary behavior as a consequence of the deficit of the executive functions (33–35). The neuropsychological dimension also links to this group of topics the studies that describe the tools for assessing frontal functions (36, 37). In detail:

- Topic 5 highlights the interest for eating behavior disorders and other forms of impulsive behaviors that mainly involve young people and find their valid explanation in the concept of personality. Attention to the afore mentioned topic produced about 250 studies.
- Topic 15 identifies the relevance, in the context of the study of personality, of the subject of measurement systems. This topic has involved over 150 research studies in the last 5 years.
- Topic 17 topic is present in more than 200 studies and expresses interest in the efficiency of higher cognitive functions in personality pathologies.
- Topic 30 is present in approximately 290 studies and expresses its interest in the emotional affective dimension in the individual's personality structure.

In the seventh group of topics, the personality is investigated in association with psychosis and post-traumatic disorders. This association stems from the implicit assumption that personality determines the terrain on which the traumatic and relational events are grafted that are capable of producing the pathology insofar as they encounter a scarcely resilient constitutional terrain. The latent concept of this group is the vision of the psychic disorder in which the personality structure and a traumatic event (usually relational) interact in generating the pathology. In this sense post- traumatic disorders, psychotic conditions and PDs are united in a single vision of the genesis of mental illness (38). In detail:

- Topic 1 is present in more than 200 studies and expresses a line of research that connects schizophrenia and obsessions to personality.
- The topic 13 expresses the link between personality functioning and stress, trauma, post-traumatic disorder, and dissociative states. Interest in this theme can be found in over 200 works.
- Topic 22 is present in more than 180 studies and expresses the interest for the study of the relational dimension of the personality.
- Topic 27 is present in little more than 180 studies and expresses the interest for borderline PD aimed at establishing characteristics and symptomatology. In the eighth group we introduced two non-related topics that concern violent criminal behaviors and chronic and degenerative organic diseases and their evolution; these are strong themes for their social and health burden for which personality is considered a determining element (39). In detail:
- Topic 6 describes the different forms of manifestation of the aggression and the close relationship between the personality and violent antisocial behavior, gender violence and sexual violence. Interest in this topic became evident in about 350 studies
- Topic 12 focuses on role of personality in the socio-health management subjects suffering from chronic or degenerative pathologies. Over 300 studies in the last 5 years have dealt with this topic.

## Discussion and Conclusion

The scientific panorama concerning the study of the impact of personality on mental health is clearly described in the eight clusters identified by their latent themes. We report them below: the conceptual revision of the personality (more or less 1,120 studies); clinical practice (more or less 950 studies); the biological cause of PDs (more or less 600 studies); development process and natural history of personality pathology (more or less 1,380 studies); typical symptoms and clinical manifestations associated with personality pathologies (more or less 1,120 studies); neuropsychological dimension of personality pathology (more or less 840 studies); diathesis-stress theory as an etiopathogenetic model of psychic disorders (more or less 760 studies); social and health impact of personality pathology (more or less 650 studies). The central element from the current scientific discussion is the evolution of the concept of PD from a categorical construct to a dimensional construct that identifies the personality structure not as a disease but as a psychic phenotype produced by the interaction between organism and environment (40). From the analysis of the data it is clear that the interest and the concrete commitment in the scientific community have been more focused, in the last five years, on the concrete aspects related to personality and mental health, in adherence with the recommendations of the international organization, with the most urgent needs expressed by patients, their families, and society as a whole (41).

Below we highlight the shortcomings that emerged in our review, indicating the desirable future directions.

In the studies that focus on the relationship between personality and mental health, the dimensional theme is still scarcely present, while the exhortation of the clinical weight of the categorical nosography prevails. The evidence-based approach, built on randomized double-blind trials, was unsatisfactory for many of the most widespread psychotherapeutic approaches and did not compensate for the expectations that had been invested in it (42). In our opinion, this area of investigation requires a commitment much greater than that described by our study, which has identified, in topic 24, just over 120 studies in this regard.

Research on the biological and genetic dimensions of personality pathologies is currently restricted to two major themes, impulsive and violent behavior, and PDs of the psychotic spectrum. The analysis of the relationship between temperament, neurobiology, and genetics in its widest sense would fill a scientifically unacceptable vacuum in the knowledge of this central aspect of human beings (37, 43, 44).

Although attention to PDs has been important for at least 50 years, there are very few follow-up studies regarding the development and evolution of PDs.

Essential questions about the differences in the pathology of personality related to gender, age, ethnic origin, relational antecedents (excluding childhood traumas that explain only part of the phenomenon), and prognosis remain unanswered (45).

The typical clinical manifestations of personality pathology, unlike what we can imagine, are not yet well defined and are often confused with some associated manifestations such as the use of drugs. For example, we still have little data on the sexual functioning or on the basic biological rhythms of individuals with PD and we do not know how to enter this information in the clinical picture and in the treatment plan (46).

A more innovative stimulus is given by research concerning neuropsychological functions and personality pathology, currently related to borderline, antisocial, and schizoid disorders. Although the link between the activity of the frontal lobes and the social functioning of the subjects has always been known, a systematic interest in this study has only been displayed in the last ten years. There is a risk in this research approach that sometimes seems to give in to the reductionist temptation to place the personality in the frontal lobes (47).

The etiology of PD is perhaps the least known element of the subject in question. The study of the interactive dynamics between body, cultural, and relational dimension is in an embryonic phase. This area of investigation needs a powerful effort to get out of the narrow scope of the stress-diathesis model and open up to circular and complex etiopathogenetic models (48).

It highlights, again, an approach to the study of personality that makes extensive use of the categorical nosography of *DSM-5* (49). The study of personality and mental health still proceeds, prevalently, according to a practical- clinical approach; too little is still moving, instead, according to an innovative research approach, but the topics analyzed to bring to light the common commitment of scholars to a new way of approaching the study of personality that can clarify a theme so complex and still lacking substantial scientific evidence.

Finally, it is necessary to recognize how the personality or some specific pathological traits underlie phenomena of social relevance (such as intra- family violence or juvenile crime), health (such as the surge of chronic cardio and cerebrovascular diseases), and dysregulation (such as affect and behavioral and interpersonal dysregulation) and at the time complicate the management of these same phenomena (50, 51). In this area, scientific research requires multidisciplinary models that integrate sociology, sports medicine, nutritional medicine, and AI technology tools (52, 53).

To implement the change in the scientific paradigm of reference, the only viable path seems to be that of a widespread and serious commitment in scientific research, as a strategy for achieving greater knowledge that can also be translated into plans for preventive interventions for personal wellbeing and public mental health.

It is hoped that the study approach presented in this review will be reflected in the academic as well as clinical, to spread a different way of conceptualizing and treating the personality according to the already existing dimensional method.

This study has the following limitations. This study did not include exploratory factor analysis (54), global network analysis (55), and number of papers by countries (56–60) as reported in other studies of LDA (61–64).

## Author Contributions

RS, GM, and NM conceived and designed the investigation. RS, DI, LuM, EM, FS, and VR conceived, designed, and performed analysis and interpretation of data. RP, VM, AM, LaM, SD'O, EG, AC, and CS worked on analysis and interpretation of the data. RS, GM, and MC drafted the manuscript. NM and MM reviewed the manuscript. All authors approved the final manuscript.

## Conflict of Interest

The authors declare that the research was conducted in the absence of any commercial or financial relationships that could be construed as a potential conflict of interest.
